# Matrix Metalloproteinase Activity in Gingival Crevicular Fluid and Periodontal Health Status in Down Syndrome Patients: A Comparative Study

**DOI:** 10.7759/cureus.40280

**Published:** 2023-06-12

**Authors:** Nizar Ahmed, Devi Arul

**Affiliations:** 1 Periodontology, Sri Ramachandra Institute of Higher Education and Research, Chennai, IND; 2 Periodontology and Implantology, Sri Ramachandra Institute of Higher Education and Research, Chennai, IND

**Keywords:** chromosomal abnormalities, trisomy 21, matrix metalloproteinase, chronic periodontitis, down’s syndrome

## Abstract

Introduction

Periodontal diseases, caused by gram-negative bacteria, often begin as gingivitis and can progress to periodontitis, characterized by inflammation extending to the periodontal ligament and alveolar bone. Individuals with Down syndrome (DS) commonly exhibit poorer oral hygiene and a higher prevalence of severe chronic periodontitis. This study aimed to identify unregulated risk factors in DS that contribute to increased periodontal breakdown.

Materials and methods

We conducted a study with 60 age-matched patients, including 20 DS patients from Balavihar Special School and 40 systemically healthy patients with and without periodontitis from Thai Moogambigai Dental College and Hospital. We collected patients' complete case histories and blood samples for evaluating matrix metalloproteinase 8 (MMP8) and matrix metalloproteinase 9 (MMP9) levels. All patients underwent nonsurgical periodontal therapy, and the samples were processed at the Central Research Laboratory at Meenakshi Ammal Dental College and Hospital. We calculated each group's mean and standard deviation and compared them using one-way analysis of variance and Kruskal-Wallis tests, followed by post hoc (Tukey honestly significant difference) multiple group comparisons. Statistical analysis was performed using Statistical Product and Service Solutions (SPSS) Statistics for Windows, Version 17.0. (Chicago: SPSS Inc.).

Results

The mean value of MMP8 in the DS group with chronic periodontitis was -18.1895, which was statistically significant (P<.001) compared to the mean value of -20.3720 in systemically healthy subjects with chronic periodontitis and -21.7120 in systemically healthy controls. Similarly, the mean value of MMP9 in the DS group with chronic periodontitis was 18.6455, which was statistically significant (P<.001) compared to the mean values of 19.8540 in systemically healthy subjects with chronic periodontitis and 25.2505 in systemically healthy controls. These findings indicate that DS subjects exhibit increased levels of pro-inflammatory cytokines MMP8 and MMP9, serving as markers for identifying periodontal disease. The mean differences in MMP8 and MMP9 in the DS group with chronic periodontitis showed highly statistically significant levels compared to both systemically healthy groups.

Conclusion

This study aimed to identify unregulated risk factors in DS that contribute to increased periodontal breakdown. Our findings revealed elevated MMP8 and MMP9 in DS patients with periodontitis, indicating an increased risk for early development of destructive forms of periodontal disease in this population. Extensive gingival tissue inflammation, bleeding on probing, increasing probing depths, loss of periodontal attachment, and alveolar bone loss are all common symptoms.

## Introduction

Periodontitis is a multifactorial disease condition that primarily affects the tooth-supporting structures of the tooth, the etiological factors include local factors like plaque microorganisms, variations in inflammatory mediators like matrix metalloproteinases (MMPs), and other cytokines. Dental plaque is a dynamic entity made up of salivary proteins and mainly bacteria, the periodontopathogens potential of each bacterial complex, especially the red bacterial complex and green bacterial complex which is primarily of gram-negative bacteria, the Down syndrome (DS) has defective host response and increased proinflammatory cytokine production which worsen the onset and the course of periodontitis and A subfamily of at least 16 zinc- and calcium-dependent proteolytic enzymes known as MMPs are responsible for mediating the breakdown of extracellular macromolecules such as collagen, fibronectin, laminin, and proteoglycan core protein. These enzymes are found in the interstitial and basement membranes of cells. The primary cell types (fibroblasts, keratinocytes, endothelial cells, and macrophages) in both inflammatory and healthy periodontal tissues can react to growth factors, cytokines, and microbial products by activating the transcription of MMP genes.

MMP inhibitors, precursor activation, substrate specificity variations, and transcriptional control of MMP genes all affect how active MMP is against extracellular matrix (ECM) substrates are regulated by four gates called regulators. The abnormal destruction of the ECM during periodontitis is a result of an imbalance between MMPs and their endogenous inhibitors. John Langdon Down initially characterized the phenotypic manifestation of individuals with circulation and coordination issues as having DS, also known as trisomy 21 (having an extra copy of chromosome), in 1866. Jerome Lejeune proposed the theory that trisomy of the 21st chromosome may result from non-disjunction (three copies of chromosome 21 instead of the usual two copies) a century later [1]. Our current study is specifically focused on DS in the south Indian population existing literature lacking sufficient data for a specific ethnicity.

This study’s objective was to investigate and compare the levels of MMPs (MMP8 and MMP9) in individuals with DS who have periodontitis (experimental group) to systemically healthy individuals with and without periodontitis (control groups). This comparison aimed to identify potential risk factors in DS that may contribute to increased periodontal breakdown and better understand the pathogenesis of periodontal disease in this population.

## Materials and methods

We conducted a case-control study that compares the levels of MMPs in individuals with DS who have periodontitis (experimental group) to systemically healthy individuals with and without periodontitis (control groups).

Study subjects and setting

In this study, we used 20 DS patients as the experimental group and 20 controls who were age- and systemically healthy-matched. Samples for the DS individuals were collected from the Balavihar School of Special Children in Kilpauk, Chennai, Tamil Nadu. From patients visiting the Department of Periodontology at the Thai Moogambigai Dental College and Hospital, Maduravoyal, Chennai, systemically healthy age-matched controls with periodontitis were selected. The study received permission from the institution's institutional ethical committee, and all study participants and the head of Balavihar School for Special Children gave their informed consent.

There were three groups of DS and control subjects: Group I (experimental) had 20 DS individuals with periodontitis, Group II (control) contained 20 systemically healthy participants with periodontitis, and Group III (control) contained 20 systemically healthy individuals without periodontitis. Calculus, bleeding on probing, and pockets were all evaluated using Community Periodontal Index for Treatment Needs scores. The study excluded those with intellectual disabilities, endocrine disorders, coronary heart disease, smokers, pan chewers, or other systemic ailments. To measure the oral hygiene state, a simple oral hygiene index was utilized. The oral hygiene index consists of two components: debris index and calculus index, index is taken in the buccal surface of 12 numerical determinations. Values from 0-3 (0 considered as no debris and calculus and 3 considered as more than 3/4th of the teeth covered with debris and calculus).

Sample collection

We collected 5 mL of blood from the median antebrachial vein in the antecubital fossa of the left arm, which was immediately transferred to the laboratory for centrifugation. Serum was obtained from the centrifuged samples. We prepared the agarose mix in a 1X-TBE (Tris-Borate-Ethylene Diamine Tetra Acetic acid) buffer using a microwave oven to achieve a clear homogeneous solution, which was cooled to 55°C before adding ethidium bromide. The solution was poured into the gel mold with combs placed and allowed to settle for 30 minutes, and then the combs were carefully removed, and the tapes were peeled off from the gel casting tray. The gel was submerged in the buffer, and electrodes were connected for a 20 mA pre-run for approximately 15 minutes. We mixed 5 µl of polymerase chain reaction mixture from each reaction tube with gel loading dye and loaded it into each well of a standard 2% agarose gel containing ethidium bromide (0.5 µg/mL). We simultaneously loaded a 100-base pair molecular marker DNA into the first lane. The power supply was turned on, and the current was adjusted to 20 mA. The gel was run until the dye reached the end of the gel, and then the gel containing complementary DNA was visualized on an ultraviolet transilluminator and subjected to densitometric scanning using a densitometer (Yercarud Biotech).

Statistical analysis

We estimated the mean and standard deviation for every experimental group and compared the mean values using a one-way analysis of variance Kruskal-Wallis, followed by a post hoc (Tukey honestly significant difference (HSD)) test. Statistical Product and Service Solutions (SPSS) Statistics for Windows, Version 17.0, was used for statistical analysis (SPSS, Inc., Chicago). A p-value < 0.05 was considered significant.

## Results

The mean value of MMP8 in the DS group with chronic periodontitis is -18.1895, which is statistically significant (p<.001) compared to the mean value of -20.3720 in systemically healthy subjects with chronic periodontitis and the mean value of -21.7120 in systemically healthy controls. This finding indicates that DS subjects exhibit increased levels of pro-inflammatory cytokines MMP8, a marker for identifying periodontal disease (Table 1, Table 2).

**Table 1:** Expression of MMP8 assessed via one-way ANOVA ANOVA: analysis of variance; MMP8: matrix metalloproteinase 8; DS: Down syndrome; SD: standard deviation; SE: standard error

Groups	N	Mean	SD	SE
Healthy	20	21.7120	1.12461	0.25147
Systemically healthy with chronic periodontitis	20	20.3720	1.60860	0.35969
DS with chronic periodontitis	20	18.1895	1.08940	0.24360
Total	60	20.0912	1.94060	0.25053

**Table 2:** Expression of MMP8 final ANOVA ANOVA: analysis of variance; MMP8: matrix metalloproteinase 8; DF: degrees of freedom

Comparison	Sum of Squares	DF	Mean Square	F	Sig.
Between groups	126.446	2	63.223	37.639	0.000
Within groups	95.743	57	1.680		
Total	222.189	59			

The mean value of MMP8 in the DS group with chronic periodontitis showed highly statistically significant levels of -1.34000 compared to -2.18250 in the systemically healthy group with chronic periodontitis and -3.52250 in the systemically healthy control group (Table 3).

**Table 3:** Post hoc tests for MMP8 levels with Tukey’s HSD *The mean difference is significant at the 0.05 level. MMP8: matrix metalloproteinase 8; DS: Down syndrome; HSD: honest significant difference

Group (I)	Group (J)	Mean Differences (I-J)	Sig.
DS with Chronic Periodontitis	Systemically healthy with Chronic Periodontitis	1.34000*	0.005
	Control		0.000
Systemically Healthy with Chronic Periodontitis	DS with Chronic Periodontitis	2.18250*	0.005
	Control		0.000
Control	DS with Chronic Periodontitis	-3.52250*	0.000
	Systemically healthy with Ch. Periodontitis		0.000

The mean value of MMP9 in the DS group with chronic periodontitis is 18.6455, which is statistically significant (p<.001) compared to the mean values of 19.8540 in systemically healthy subjects with chronic periodontitis and 25.2505 in systemically healthy controls. This result indicates that DS subjects exhibit increased levels of pro-inflammatory cytokines MMP9, which, like MMP8, is a marker for identifying periodontal disease (Table 4, Table 5).

**Table 4:** Expression of MMP9 via one-way ANOVA ANOVA: analysis of variance; MMP9: matrix metalloproteinase 9; DS: Down syndrome; SD: standard deviation; SE: standard error

Groups	N	Mean	SD	SE
Healthy	20	25.2505	1.22334	0.27355
Systemically healthy with chronic periodontitis	20	19.8540	1.72163	0.38497
DS with chronic periodontitis	20	18.6455	1.07349	0.24004
Total	60	21.2500	3.19261	0.41216

**Table 5:** Expression of MMP9 final ANOVA ANOVA: analysis of variance; MMP9: matrix metalloproteinase 9; DF: degrees of freedom

Comparison	Sum of Squares	DF	Mean Square	F	Sig.
Between groups	494.725	2	247.362	132.210	0.000
Within groups	106.646	57	1.871		
Total	601.371	59			

The mean difference in MMP9 in the DS group with chronic periodontitis showed highly statistically significant levels of 5.39650 compared to 1.20850 in the systemically healthy group with chronic periodontitis and -6.60500 in the systemically healthy control group (Table 6).

**Table 6:** Post hoc tests for MMP9 levels with Tukey’s HSD *The mean difference is significant at the 0.05 level. MMP8: matrix metalloproteinase 8; DS: Down syndrome; HSD: honest significant difference

Group (I)	Group (J)	Mean Differences (I-J)	Sig.
DS with Chronic Periodontitis	Systemically healthy with Chronic Periodontitis	5.39650*	0.000
	Control		0.000
Systemically Healthy with Chronic Periodontitis	DS with Ch. Periodontitis	1.20850*	0.000
	Control		0.019
Control	DS with Chronic Periodontitis	-6.60500*	0.000
	Systemically healthy with Chronic Periodontitis		0.019

## Discussion

One of the most prevalent genetic disorders, DS has a very unpredictable prognosis [2]. People who have DS display certain orofacial features that are connected with the syndrome and are more likely to develop oral diseases including diastema, bruxism, mouth breathing, macroglossia, delayed tooth eruption, missing and deformed teeth, microdontia, and periodontal disease [3]. Among these, a high susceptibility and prevalence of periodontitis have been reported in DS patients [4]. However, the underlying reasons for this high susceptibility and prevalence remain unclear.

MMPs, also known as matrixes, are a critical family of metal-dependent endopeptidases that are involved in the turnover and breakdown of ECM components as well, which are important in development and morphogenesis. Multiple factors influence the expression of the 28 matrixes genes in human transcription, including inflammatory cytokines, growth factors, hormones, and cell-cell as well as cell-matrix interactions. MMPs are activated by precursor zymogens and inhibited by endogenous inhibitors such as tissue inhibitors of metalloproteinases (TIMPs). As a result, harmony between MMPs and TIMPs is important for final ECM remodeling [5].

MMP activity against ECM substrates is regulated by four gates called regulators [2]. These include (a) cytokines and growth factors that induce MMPs; (b) hormonal regulation; (c) cell shape and cell-substrate adhesion; and (d) second messenger signaling. Additionally, precursor activation, differences in substrate specificity, and MMP inhibitors all serve as transcription factors for MMP gene regulation.

Several studies have investigated the role of MMPs in periodontal disease. Gonçalves et al. [6] evaluated salivary MMP2 and MMP9 levels in 16 patients and discovered insignificant variations, indicating that salivary levels are a poor diagnostic of periodontal disease activity. Rai et al. [7] assessed 53 subjects and found that salivary MMP8 and gingival crevicular fluid (GCF) MMP2 and MMP9 levels were significantly higher in periodontitis patients. The results of this research indicate the importance of MMPs in the jeopardizing processes of periodontal disease.

To understand the susceptibility of periodontal disease in DS patients, we assessed serum MMP levels. MMPs are proteolytic enzymes released from various cell types present in the lesion, including macrophages, leukocytes, fibroblasts, or other resident cells [8]. MMP activity is regulated at multiple levels, including transcriptional, post-transcriptional, and post-translational. Generally, MMPs increase with inflammation and disease activity [6], and their detection has been suggested as a possible host response biomarker of periodontal disease [9]. Interestingly, the role of individual MMPs in periodontal disease progression may differ.

Recent studies have shown increased MMP2, MMP3, MMP8, and MMP9 in GCF and an altered relationship between MMP8 and TIMP2 in subjects with DS compared to controls [10,11]. Higher immune reactivity of MMP8 and MMP9 has also been demonstrated in the saliva of subjects with DS when compared to healthy controls [12]. In this study, we assessed MMP8 (collagenase 2) and MMP9 (gelatinase b) levels in DS individuals with chronic periodontitis to further confirm the release of pro-inflammatory cytokines by specific microorganisms. DS periodontitis patients showed a positive correlation with highly statistically significant values (p< .000) and increased MMP8 and MMP9. The results of our study are consistent with those of Yamazaki-Kubota et al. [10], who concluded that there is a significant increase in MMP8 levels.

Defects and dysfunction of bactericidal capacities have been described by Izumi et al. [13], which may affect MMP8 and MMP9 in DS patients. Our findings suggest that increased MMP8 and MMP9 in DS periodontitis patients could contribute to their heightened susceptibility to periodontal disease.

The study had several important limitations. First, the sample size was relatively small, including only 20 DS subjects and 40 age-matched controls, which may limit the generalizability of the findings. Second, the cross-sectional design does not establish causality or examine the progression of the periodontal disease over time, necessitating longitudinal or interventional studies better to understand the relationship between DS and periodontal disease. There was a lack of comprehensive information on the participants' oral hygiene habits, which could potentially influence periodontal disease progression. Potential confounding factors such as socioeconomic status, access to dental care, or other environmental factors may not have been fully accounted for. The study relied on serum levels of MMPs, which may not accurately reflect the local inflammatory response in the periodontal tissues; GCF or salivary markers measurements might provide more relevant information about periodontal disease activity. Our study did not investigate the presence or levels of specific periodontal pathogens in the participants, which could have helped clarify the role of different microorganisms in periodontal disease progression among DS subjects. Finally, the generalizability of the findings may be limited since the study was conducted in a specific geographic location and might not be applicable to other populations with different ethnic backgrounds or environmental factors.

## Conclusions

Our study provides evidence for the role of MMPs in the increased susceptibility of DS patients to periodontal disease. Further research is needed to explore the precise mechanisms underlying this association and to develop targeted therapeutic interventions for DS patients suffering from periodontal disease. Understanding the relationship between DS, MMPs, and periodontal disease, it may be possible to develop new strategies to mitigate the risk of periodontal disease in individuals with DS and improve their oral health. As many DS children are institutionalized, oral hygiene instructions should be provided to their caretakers. These students should undergo periodic dental screening to prevent future periodontal disease progression. Additionally, DS students should be encouraged to maintain their daily oral hygiene independently.
